# Evidence-Based Reporting in Preimplantation Genetic Testing (PGT)

**DOI:** 10.3390/genes16091083

**Published:** 2025-09-15

**Authors:** Maurizio Poli, Ludovica Picchetta, Laura Siciliani, Antonio Capalbo

**Affiliations:** 1 Reproductive Genetics, Juno Genetics, 00188 Rome, Italy; 2 Biology Department, University of Rome Tor Vergata, 00133 Rome, Italy; 3 Health Research Institute La Fe, IVI Foundation, 46026 Valencia, Spain; 4 Center for Advanced Studies and Technology CAST, “G. D’Annunzio” University of Chieti-Pescara, 66100 Chieti, Italy

**Keywords:** PGT, embryo, reporting, validation

## Abstract

Preimplantation genetic testing (PGT) reports play a decisive role in determining the fate of IVF-generated embryos. The identification of a chromosomal or genetic abnormality that could impact the health of the resulting newborn often leads to embryo disposal or indefinite storage in cryogenic containers. As a growing proportion of IVF cycles include PGT assessment, greater scrutiny is being placed on its clinical validity. Initially developed to detect monogenic disorders (PGT-M) and later expanded to identify full chromosomal aneuploidies, PGT is primarily used to identify embryos unlikely to implant (aneuploid), those that would lead to miscarriage, or those causing chromosomal syndromes or monogenic conditions. Advancements in genetic analysis now allow for the assessment of more complex traits and chromosomal features from a trophectoderm biopsy, including segmental aneuploidies, chromosomal mosaicism, and polygenic conditions. However, as technology pushes the limits of biological resolution, questions arise regarding the accuracy, clinical utility, and representativeness of these findings for the entire embryo. This article reviews the gold standards for validating clinical findings and reporting strategies, aiming to maximize diagnostic utility while minimizing false positives towards appropriately defined reproductive outcomes and phenotypes.

## 1. Introduction

Preimplantation genetic testing (PGT) is an advanced reproductive technology used to assess in vitro fertilization (IVF)-generated embryos for genetic and chromosomal abnormalities before implantation. The primary goal is to identify embryos carrying genetic defects and, through their deselection from treatment, drastically reduce the risk of miscarriage and transmission of genetic disorders, while improving implantation rates per transfer. PGT is further categorized based on the type of genetic abnormality investigated: monogenic conditions (PGT-M), structural rearrangements (PGT-SR), aneuploidies (PGT-A), and, more recently, multifactorial conditions through polygenic embryo screening (PES, also described as PGT-P). These different analytical strategies are increasingly integrated, allowing multiple assessments in a single analysis.

Evidence-based PGT findings are crucial for providing accurate, reproducible, and clinically actionable results, ensuring that embryo transfer decisions are based on reliable scientific data. Robust validation of PGT methodologies prevents the transfer of affected embryos that are categorized as normal (i.e., false negatives), which would reduce treatment performance by failing to produce a healthy pregnancy either through failed implantation, miscarriage, or birth of a newborn carrying a genetic condition which the embryo was tested for. On the other hand, overall treatment potential can also be impacted by the wastage of embryos deemed abnormal when they are actually false positives. While comprehensive evidence is available for the capability of validated PGT programs to detect monogenic conditions and uniform whole-chromosome aneuploidies [1,2,3], the reliability of findings involving more recent TE biopsy and NGS-enabled parameters, such as mosaicism, have long been under debate. Similarly, the reliability of polygene risk scores (PRS)-based PGT has been challenged due to the relative nature of the assessment and the limited clinical gain that this type of selection might have on an already limited cohort of available embryos.

Standardized guidelines for reporting PGT results are essential for the improvement of PGT practice and overall clinical outcomes, as well as for facilitating patient counseling and decision-making over the risks associated with embryo transfer.

This review explores key aspects of evidence-based reporting, including the identification and deselection of uniform aneuploidies in TE biopsies, the biological origins and clinical significance of mosaicism, the management of segmental aneuploidies, and additional genetic features detected through genotyping.

## 2. The Analytical Substrate for PGT

Prior to the advancement of culture media and incubation systems capable of reliably supporting embryo development to the blastocyst stage, embryo biopsy was routinely performed at the cleavage stage, when the embryo consisted of approximately 6 to 10 cells. At that time, the procedure involved the removal of one or two blastomeres, representing a substantial proportion (12% to 25%) of the total embryo. This approach posed significant risks, as the removal of such a large fraction of undifferentiated cells disrupted embryonic integrity and potentially interfered with lineage allocation, which could not be assessed at that stage. Consequently, cleavage-stage biopsy was associated with reduced survival rates and lower overall efficiency of PGT [4].

Additionally, the limited cellular material available for analysis resulted in high rates of amplification failure and inconclusive or unreliable results. The transition to blastocyst-stage biopsy, made possible by improvements in extended embryo culture in the late 2000s, marked a significant advancement. Blastocyst biopsy is typically performed on day 5, 6, or 7 post-insemination and involves the extraction of 5–8 trophectoderm (TE) cells from an embryo comprising over 100 cells, thereby removing only 5–8% of the total mass [3,5]. This dramatically reduces the relative impact on embryonic development compared to the earlier cleavage-stage approach.

Furthermore, by the blastocyst stage, the embryo has undergone initial lineage segregation, distinguishing the inner cell mass (ICM), which gives rise to the fetus, from the trophectoderm, which contributes to extraembryonic tissues. As a result, sampling from the TE is thought to have less direct impact on embryonic potential [4], while still providing a robust substrate for genomic analysis and optimal cryosurvival rates.

Although double TE biopsies have been explored in cases of inconclusive results, single biopsy remains the standard to minimize procedural stress and avoid additional cryopreservation cycles [6]. However, given that the entire chromosomal or genetic profile of the embryo must be inferred from a limited cell sample, careful attention must be paid to sampling bias and technical variability, especially when attempting to detect non-uniform chromosomal abnormalities, such as those occurring following post-zygotic mitotic errors leading to mosaicism.

More recently, with the growing adoption of PGT and an increasing number of embryos undergoing biopsy, concerns about the potential impact of biopsy on embryo viability have spurred interest in non-invasive or less invasive alternatives. These include non-invasive PGT (niPGT) techniques that analyze cell-free DNA (cfDNA) released into the spent culture media or blastocoel fluid.

While niPGT offers attractive features, such as minimal embryo manipulation and reduced technical demands, its clinical implementation is currently limited by substantial analytical challenges. The amount of cfDNA released by embryos is highly variable and not controllable, leading to inconsistent results. Furthermore, external contamination is more difficult to detect and eliminate in these settings, compromising the reliability of the analysis.

To date, niPGT approaches have been explored primarily in the context of PGT-A, but they still lack sufficient validation for any clinical use. Their diagnostic accuracy, reproducibility, and predictive value remain under investigation, and, at present, niPGT is not regarded as a replacement for established and validated biopsy-based techniques and should be considered only in research contexts.

## 3. The Objective of PGT-M: Identification and Deselection of Embryos Carrying Pathogenic Alleles

PGT-M is employed in cases where the prospective parents are found to be at increased risk of having a child affected by a specific genetic condition, either during family history analysis or during preconception carrier screening. PGT-M was first successfully implemented in humans in 1990 [7]. In this initial application, embryos from patients carrying X-linked recessive disorders were genetically analyzed, allowing for the selection and transfer of female embryos to prevent the transmission of the condition. This approach effectively avoided implanting male embryos, which had a 50% likelihood of inheriting and developing the disorder.

Since its introduction, PGT-M has evolved rapidly, with increasing clinical applications and technological advancements. The PGT Consortium of the European Society of Human Reproduction and Embryology (ESHRE) has monitored and documented data on these procedures. According to its most recent report, 35% of all PGT procedures were conducted specifically for monogenic disorders [8]. At present, diagnostic protocols exist for over 200 monogenic conditions, and this number continues to expand as genetic testing techniques advance [9].

From a technical perspective, PGT-M is predominantly conducted using three molecular techniques: targeted multiplex PCR, single nucleotide polymorphism (SNP) arrays analysis, and next-generation sequencing (NGS) [10,11,12,13].

Targeted multiplex PCR is employed to amplify the specific gene region of interest. The amplified DNA can then be examined using methods such as Sanger sequencing, qPCR-based genotyping, or mini-sequencing to detect the presence of the mutation or pathological variant. This strategy is typically reinforced by analyzing linked informative markers, which are polymorphic genetic elements located near the mutation site (typically within 1–2 Mb). These markers, which include short tandem repeats (STRs) and single nucleotide polymorphisms (SNPs), co-segregate with the target locus, enabling the identification of the corresponding haplotype through linkage analysis. To perform this analysis, reference samples from both parents and, ideally, an affected family member are required to build a haplotype map and test the proband.

On the other hand, SNP-array-based PGT-M is a technique that relies on the prior characterization of the SNP profiles within the parental genomes and reference samples of affected family members, and the identification of the haplotype associated with the pathogenic variant. By analyzing the inheritance pattern of SNPs in the embryo, this approach enables the determination of whether the embryo has inherited the disease-associated haplotype without direct mutation identification [14].

NGS-based approaches for PGT-M involve the targeted amplification of the relevant gene region, facilitating the simultaneous analysis of both the pathogenic mutation and its associated flanking markers [15]. This comprehensive evaluation enhances the accuracy of the genetic diagnosis.

Across these methodologies, the accuracy rates are consistently exceeding 99%, with a misdiagnosis rate below 0.1%, highlighting the high reliability of PGT-M technologies [15,16]. Across all the PGT approaches, PGT-M is the most widely accepted as it provides a solid mean to avoid pregnancies carrying an inheritable pathological condition.

Despite its broad and generally effective application, PGT-M presents both technical and clinical limitations that may compromise its utility in specific contexts.

From a technical standpoint, PGT-M struggles with the accurate detection of mitochondrial disorders and trinucleotide repeat expansion diseases. In mitochondrial disorders, the precise quantification of heteroplasmy (i.e., the proportion of mutated to wild-type mitochondrial DNA) is difficult, limiting the ability to reliably predict disease severity. In repeat expansion conditions, such as Huntington’s disease, fragile X syndrome, and myotonic dystrophy, clinical utility can be reduced because not all methods allow characterization of repeats length in embryo biopsies. In these cases, it is common practice to inform patients about the presence or absence of the at-risk genotype to facilitate clinical decision-making.

Clinically, the value of PGT-M may be reduced in cases involving ambiguous indications, such as variants with low penetrance, mild phenotypes, or variable expressivity. Examples include certain *CFTR* mutations, *GJB2*-related hearing loss, or genes with complex inheritance patterns, such as *NEMO* (*IKBKG*) (i.e., an X-linked gene where female expression depends on skewed X-inactivation and male viability may require mosaicism or hypomorphic mutations). In these instances, defining embryo suitability for transfer becomes ethically and diagnostically complex and a substantial level of variability exists in the practice of PGT-M across countries where different regulations and legal frameworks are in place to support the appropriateness of indications for PGT-M.

Moreover, applying binary classifications, such as “affected” or “unaffected”, may oversimplify the genetic risk in cases of incomplete penetrance (e.g., *BRCA1/2*, *FSHD*), variable expressivity (e.g., Neurofibromatosis type 1, Marfan syndrome), or carrier status with potential for clinical expression (e.g., certain *CFTR* or *PAH* variants). Although PGT-M is already being performed in cases such as the ones described, in these scenarios, specific genotype reporting may be warranted. Such reporting should always be accompanied by thorough pre- and post-test counseling conducted by a qualified genetic counselor to help the couple understand the limitation of the test and the implications of the findings. This approach would offer greater clinical transparency and supports more informed decision-making.

## 4. The Objective of PGT-SR: Identification and Deselection of Unbalanced Embryos

Structural chromosomal rearrangements affect only a portion of a chromosome and can be classified as either unbalanced, typically detected as segmental gains or losses, or balanced, in which no net gain or loss of DNA occurs. Balanced rearrangements, such as translocations and inversions, are often identified in individuals with otherwise normal phenotypes, particularly during fertility assessments. These rearrangements can be associated with subfertility due to an increased risk of generating unbalanced gametes during meiosis. The incidence of balanced structural chromosomal abnormalities is approximately 0.7% in the general population, but this increases to 2.2%, 4.8%, and 5.2% following one, two, and three miscarriages, respectively [17]. These individuals are typically identified through pre-conceptional karyotyping, and, in such cases, preimplantation genetic testing for structural rearrangements (PGT-SR) is recommended. A key consideration with PGT-SR is the variability in platform resolution across different testing technologies. Therefore, before proceeding, a feasibility assessment must be conducted by the genetic laboratory. This assessment evaluates whether the platform can detect the specific unbalanced chromosomal fragments indicated by the karyotype. In some cases, the rearrangement may involve regions too small, or regions not covered by the platform’s amplification process (e.g., telomeric regions), rendering the test unfeasible. PGT-SR results are typically reported as either “balanced” or “unbalanced”, since most technologies cannot distinguish between normal and balanced embryos. However, newer technologies, originally based on massive parallel sequencing and long-read sequencing, have been developed, allowing for the distinction between normal embryos and those carrying a balanced rearrangement. More recently, genotyping and haplotyping with phasing analysis have enabled universal PGT-SR, which does not require prior identification of breakpoints and is no longer dependent on the type of rearrangement [18]. While these advances provide broader applicability, they introduce ethical considerations. Embryos with a balanced rearrangement, despite being otherwise viable and sometimes exhibiting superior morphology, may be deprioritized simply because they carry a structural variant. Currently, no formal guidelines exist regarding how these reports should be produced and presented to the patients. This underscores the critical importance of comprehensive genetic counseling, both before and after testing. Pre-test counseling should ensure couples fully understand the implications and limitations of PGT-SR, while post-test counseling must provide clear interpretation of results, to support informed decision-making.

## 5. The Objective of PGT-A: Identification and Deselection of Embryos Carrying Uniform/Meiotic Aneuploidies

PGT-A primarily aims to identify embryos with numerical chromosomal abnormalities that arise due to errors during meiosis (i.e., aneuploidies). Meiotic aneuploidies are inherited from the gametes and typically result in whole chromosomal gains or losses. Their origin is mainly maternal, due to the age-related increase in faulty chromosome separation processes which lead to genetic imbalances in the mature oocyte [19]. Gamete meiosis-derived aneuploidies are expected to uniformly affect all cells of the ensuing embryo.

Uniform whole-chromosome aneuploidies are almost always incompatible with implantation, or lead to miscarriage and congenital disorders (e.g., Down syndrome—trisomy 21, or Turner syndrome—monosomy X).

In medical diagnostics, positive predictive value (PPV) is essential for determining how likely a positive test result corresponds to a true affected case. However, in the context of PGT-A, calculating PPV conventionally is challenging since many aneuploid embryos fail to implant and cannot be independently verified. As a result, PPV in PGT-A is interpreted differently, referring to the likelihood that an aneuploid result predicts embryonic developmental failure or, in rare cases, the persistence of chromosomal abnormalities in viable pregnancies (e.g., trisomies 13, 18, 21, and sex chromosome aneuploidies). Since most aneuploidies detected through PGT-A are not viable beyond the first trimester, reproductive lethality is often used as a proxy for clinical prognostic value.

The PPV of PGT-A is defined as the proportion of transferred aneuploid embryos that either fail to implant/develop or result in confirmed aneuploid pregnancies. Conversely, negative predictive value (NPV) refers to the probability that an embryo classified as euploid is truly free of aneuploidies detectable within the specific limitations of the technology employed.

While NPV data are naturally collected in clinical settings, since euploid embryos are routinely transferred and their follow up monitored, PPV assessments require controlled experimental studies where aneuploid embryos are transferred with patient consent or where blinded testing is conducted. There are currently five studies where, in addition to putative mosaic embryos, uniformly aneuploid embryos were also transferred to patients [1,2,20,21,22]. As summarized by Capalbo and colleagues in 2022, out of 353 transfers, 51 led to embryo implantation (14.4%), and, out of which, 44 resulted in miscarriage (86.3%) [23]. Overall, 98% of transfers performed resulted in embryo lethality (n = 346/353), confirming exceptionally high PPV of PGT when employed to detect uniform aneuploidies. To note, four of the seven pregnancies that resulted in live birth derived from the only study carried out in 2012, prior to NGS application [1]. Nonetheless, in the most relevant of these studies, a multicentre, prospective, blinded, and non-selection design was employed by Tiegs and colleagues to evaluate the predictive value of aneuploidy diagnosis using NGS-based PGT-A [2]. Here, the outcomes from 102 aneuploid embryo transfer was compared with 312 transfers of euploid embryos in a matched population. In the euploid group, 202/312 transfers led to sustained pregnancy or healthy delivery, while, in the experimental group, 0/102 embryos progressed to live birth, clearly demonstrating the clinical utility of modern PGT applications.

These studies provide proof-of-principle that PGT-A may achieve high predictive values in carefully controlled settings; however, they do not establish universal safety or efficacy across all laboratories, technologies, or patient populations. Because predictive values are prevalence and protocol dependent, each center must validate its own sensitivity, specificity, PPV, and NPV before clinical implementation.

One possible way to validate PGT-A reliability involves multiple biopsies, including cells from the inner cell mass, followed by blinded aneuploidy testing.

A 2020 review of re-biopsy studies confirmed that the reliability of PGT-A results depends on various factors, such as the testing technology and the criteria employed for assessing concordance rates [24]. The study found that when embryos were classified as euploid or uniformly aneuploid, follow-up testing confirmed these classifications in over 95% of cases, demonstrating high accuracy and reproducibility for uniform chromosomal abnormalities. However, these consistent confirmation rates do not apply when NGS quantitative analysis is employed to detect mosaicism and segmental abnormalities.

In summary, both analytical and clinical validation are essential pre-requisites for the implementation of a PGT platform in a clinical setting and derive evidence-based criteria for aneuploidy reporting in a clinical setting. While analytical validation ensures that the platform is accurate and reproducible under laboratory conditions, a subsequent clinical validation is fundamental to establishing the real-word effectiveness and reliability of the platform for actual clinical samples. Analytical validations are often conducted using both euploid and aneuploid cell lines, including mixtures at varying ratios, particularly for validating intermediate chromosomal copy numbers (ICN). However, unlike standardized cell lines, clinical samples can vary significantly, both in terms of quality and in terms of number of biopsied cells. As a result, a clinical validation is the only reliable strategy to test the platform under the complex and variable scenarios encountered in routine IVF settings. Clinical validations are needed to complement the analytical validations in order to ensure that high performances of the platform are maintained even under real-world conditions. PGT-A applications should always be validated prior to clinical implementation via a non-selection trial. These trials are, in fact, specifically designed to minimize the risk of population selection biases that are, on the contrary, often present in retrospective studies and can distort the results due to confounding factor such as patient prognosis influencing the outcome under investigation.

## 6. Intermediate Chromosomal Values and Mosaicism

Next-generation sequencing (NGS) has significantly improved the diagnostic capabilities of preimplantation genetic testing (PGT) by enhancing both analytical power and throughput while reducing costs. The combination of multicellular specimens derived from trophectoderm (TE) biopsies and the increased resolution of NGS allows for the detection of additional genomic features, including ICNs, which might indicate chromosomal mosaicism in the biopsy or the presence of technical artifacts.

Embryo mosaicism refers to the presence of two or more genetically distinct cell populations within the same embryo, arising from mitotic errors in blastomeres or post-fertilization embryonic cells. When an embryo exhibits a mix of euploid and aneuploid cells, characterized by intermediate chromosomal numbers (between 1–2 and 2–3), different reporting strategies have been proposed. One common approach classifies mosaicism based on ICN and infers from this the proportion of aneuploid cells in the biopsy, distinguishing between low-grade (deviation lower than <50%) and high-grade (deviation higher than >50%) mosaicism. Alternative strategies define low-grade mosaicism as 30–50% and high-grade as 50–70%, while additional strategies may extend the thresholds to 20–50% (low-grade) and 50–80% (high-grade), ultimately increasing the number of embryos classified as mosaic [25].

One of the main challenges of mosaic embryo diagnosis is the uncertainty of the risks associated with the finding and their clinical implications, which ultimately lead to an increased likelihood of embryo abandonment. Additionally, the classification of mosaicism carries inherent unreliability due to both biological and technical factors. Technically, ICN values may result from signal noise, incomplete specimens, or variability in whole genome amplification. Biologically, while the biopsied sample may not accurately reflect the entire embryo, the reliability of mosaicism rate estimates (normal vs. abnormal cells) is also uncertain, as the exact number of cells biopsied is unknown, introducing potential discrepancies in the assessment of aneuploidy. False positive mosaicism reporting can also be observed following contamination of the test tube with exogenous DNA and when an extra or missing chromosome is present in a polyploid embryo. Thus, lineage-based reporting approaches face limitations in reproducibility and consistency. Accordingly, a recent GPR paper from ESHRE has suggested that whenever an ICN is detected in a TE biopsy this should not be reported as definitive evidence of mosaicism. This type of findings of uncertain clinical significance can either be ignored or reported as “putative” or “suggestive of” mosaicism [26].

Retrospective studies indicate that embryos displaying low-grade ICN in the biopsy often implant and develop similarly to euploid embryos, whereas embryos showing high-grade ICN in the biopsy are associated with increased miscarriage rates and implantation failures [27,28]. Moreover, in a large prospective non-selection study including almost 900 transfers, embryos displaying medium and low-grade ICN in the biopsy were classified as euploid and transferred. Here, not only live-birth and miscarriage rates were comparable across groups, but also gestational, obstetrical, and neonatal parameters were similar, with no tested cases from the putative mosaic groups presenting abnormal karyotype or uniparental disomy configuration at post-natal follow-up [29]. However, it is likely that many euploid embryos are misclassified as low-grade mosaic, and high-grade mosaics may actually be uniformly aneuploid as demonstrated by Popa et al., 2025 [30]. Variations in analytical output are likely to stem from technical deviations in DNA amplification and processing rather than true biological mosaicism. For these reasons, reporting of embryo mosaicism in PGT is an extremely delicate topic and should be carefully managed in order to maximize clinical outcomes while minimizing negative consequences. Currently available prospective blinded non-selection studies showed that low-grade ICN identified through low-pass NGS provided no clinical utility as they resulted in comparable clinical outcomes to uniformly euploid [29,31].

Because embryos displaying varying degrees of ICN in the biopsy have produced mixed clinical outcomes, the clinical implications of mosaicism remain a subject of ongoing research. It is important that every technology used in clinical practice undergoes prospective blinded studies to properly define reporting criteria and predictive values of ICNs detected in the biopsy.

Improved reporting standards are needed to provide clinicians and patients with clear guidance on the clinical utility of mosaic reporting in PGT. To facilitate this task, the application of novel qualitative methodologies (i.e., NGS-based genotyping) to complement standard quantitative NGS, can provide additional data for discriminating abnormalities of meiotic and mitotic origin (see further).

## 7. Management of Segmental Aneuploidies

Segmental aneuploidies involve structural chromosomal alterations, specifically deletions and duplications, that affect only part of a chromosome rather than the entire chromosome. These aberrations may arise from faulty repairs of double-strand breaks, erroneous chromosomal breakage correction, or inherited structural abnormalities from a parent, and they can have variable clinical consequences depending on the genomic regions involved (e.g., gene content).

Current evidence shows that segmental aneuploidies are quite common in preimplantation embryos, with an average incidence of 8.51%, highly variable depending on the resolution of the technology that is utilized [32]. Segmental aneuploidies differ significantly from whole-chromosome aneuploidies in several ways. Most importantly, segmental aneuploidies are not associated with maternal age and typically arise during the embryonic mitotic divisions [33,34]. This mitotic origin presents a key challenge in PGT-A reporting. Indeed, a mitotic origin should, by definition, imply a mosaicism in the embryo, meaning that not all cells within the embryo carry the abnormality, therefore reducing the PPV of PGT-A for segmental aneuploidies. This hypothesis is supported by evidence from studies where multifocal biopsies showed a low concordance rate, averaging around 30%, for segmental aneuploidies between subsequent biopsies of the same embryo [32,35,36]. Nevertheless, a low concordance rate alone does not definitively indicate the presence of a true biological mosaicism, that can only be accurately inferred if reciprocal abnormalities are detected (i.e., a deletion and a duplication of the same fragment). Another potential explanation for the lower concordance rate of segmental aneuploidies compared to whole chromosome aneuploidies might be technical artifacts of PGT-A such as analytical noise or sampling bias due to the small number of biopsied cells. The potential of both mosaicism and technical artifacts is also supported by evidence from embryo transfer outcomes, proving that segmental aneuploid embryos do have a reproductive potential, albeit reduced compared to euploid embryos. Unfortunately, the data is still quite scarce and mainly limited to ICN segmental aneuploidies [2,37,38,39,40], with only one study so far that has investigated the impact of full segmental aneuploidies on embryo transfers [41]. Regardless of the type of investigation, all studies report live-births, and some also tested pre- and post-natally, of healthy new-borns. Even the only study on full segmental aneuploid embryos, by Besser and colleagues, reports a 24% live birth rate for these embryos, with none of the tested pregnancies resulting positive for the abnormality [41]. Given that segmental aneuploid embryos, unlike those with whole chromosome aneuploidies, retain some reproductive potential, there is ongoing debate within the scientific community regarding how these embryos should be reported and whether they should be considered for transfer. One of the main difficulties in interpreting segmental aneuploidies detected during PGT-A is the lack of the possibility to make genotype–phenotype correlations, making it extremely difficult to predict their potential impact on later stages of development. Another critical factor is the uncertainty about whether a segmental aneuploidy identified in a single trophectoderm biopsy also affects the ICM, which ultimately forms the fetus. In this regard, according to Girardi et al., the best current predictor of ICM status in these cases is a re-biopsy strategy [35]. A negative result on re-biopsy, due to a potential mosaicism or technical artifact in the first biopsy, increases the likelihood of the ICM being normal, thereby improving the chances of a healthy pregnancy if the embryo is transferred. Further insights into the clinical relevance of segmental aneuploidies are also expected to arise with the more prominent use of genotyping-based technologies, which will be further discussed in the following section. Genotyping, when combined with parental DNA, can also help distinguish cell division of origin in a single biopsy. This distinction may reveal itself to be fundamental to assess the different impact of mitotic and meiotic origin segmental aneuploidies, with an expected lesser negative impact on development of the firsts compared to the latter.

In the context of an evidence-based framework for PGT, reporting of segmental aneuploidies also requires validation through non-selection studies. Current evidence shows that around half of them have a meiotic origin or ICM involvement, and the transfer of embryos displaying a full segmental aneuploidy in the clinical TE biopsy is associated with a lower reproductive potential [41]. However, considering that available evidence from embryo re-biopsy studies and clinical experience suggests that embryos with segmental aneuploidies still retain substantial reproductive potential, thorough genetic counseling is essential to inform decision-making on the clinical utilization of segmental abnormal embryos.

## 8. Additional Genetic Features Identified Through Genotyping

Conventional low-pass NGS approaches have limited resolution when it comes to distinguishing the underlying origin of subtle variations observed in the sequencing data.

In contrast, genotyping-based technologies, particularly those that analyze SNPs across the genome, offer qualitative insights into both genetic variants and the chromosomal status of the sample. The parallel application of quantitative and genotyping approaches can reveal additional information regarding both the health of the embryo and the quality of the specimen and its associated findings. For example, this combined approach can validate the presence of a normal bi-parental diploid status, while also detecting all forms of haploidy (23 chromosomes) and triploidy (69 chromosomes), as well as some types of uniparental diploidy (46 chromosome inherited from a single gamete), that would otherwise go undetected with technologies relying on copy number only [42,43]. Additionally, the use of genotyping can provide valuable quality control measures like fingerprinting analysis to verify genetic relationships, like siblingship, and the detection of contamination from exogenous DNA in the biopsy specimen that may both hinder an accurate diagnosis [44]. SNP analysis can now be performed using both SNP array-based platforms and NGS-based methods, enabling the simultaneous assessment of multiple genomic regions associated with various traits or conditions.

In particular, SNP profiling enables the determination of the biological origin of chromosomal abnormalities, distinguishing between those that arise during meiosis and those that likely occur post-zygotically during mitosis and providing critical insights for reporting strategies in PGT-A [30,45].

This capability is especially valuable for interpreting ICN. For instance, if a suspected ICN is found to originate from a meiotic error, it strongly suggests a uniform aneuploidy and effectively rules out mosaicism. Although this approach is technically feasible and likely to offer clinical value, only a limited number of reports have addressed it to date (Popa et al., 2025 [30]). Currently, robust clinical validation is still lacking. Ideally, such validation should be carried out using non-selection strategies to minimize potential biases, while ensuring that the origin of the cell division error remains concealed until after the embryo has been transferred into the uterus.

Moreover, genomic profiling has opened the door to identifying genetic predispositions to complex diseases that are under polygenic control, such as diabetes or cardiovascular disorders [46]. Polygenic risk scores (PRS) are calculated based on genome-wide association studies (GWAS) conducted on large populations. While the potential to predict the risk of developing such conditions is scientifically appealing, the clinical utility of these models remains debated. One of the main limitations of polygenic embryo screening (PES) consists of current evidence that many genes involved in PRS contribute to multiple, unrelated phenotypes (i.e., pleiotropic genes). For example, a variant that lowers the risk for type 2 diabetes might simultaneously increase the risk for autoimmune disorders or impact fertility, neurodevelopment, or psychiatric traits. As a result, pleiotropy of genes makes risk-reducing embryo selection complex and potentially counterproductive, as choosing an embryo based on a lower risk for one condition might inadvertently raise the risk for another. Moreover, estimating the likelihood of developing a polygenic condition based solely on genomic data oversimplifies the multifactorial nature of these diseases, which result from a complex, context-dependent interplay between genetic background and environmental factors, ultimately bringing into question the predictive accuracy and real-world relevance of such models in embryo selection.

By identifying these anomalies, genotyping adds an additional layer of quality control, helping to ensure that reported findings more accurately reflect the true chromosomal constitution of the embryo. Given the diversity of technologies available in the genetic testing landscape, genotyping alone does not guarantee the presence of clinically validated algorithms for preimplantation genetic testing (PGT). It is essential that genotyping-based algorithms undergo rigorous validation for all analytical and clinical parameters reported in the context of PGT. This validation must be specific to both the intended clinical application and the specific technological platform being used, to ensure accuracy, reliability, and clinical utility of the results.

## 9. Challenges and Limitations in Evidence-Based Reporting

The use of diverse technologies with varying levels of analytical and clinical validation across platforms leads to differences in both the accuracy and granularity of genetic testing results. While current NGS-based strategies offer reliable detection of uniform aneuploidies and monogenic disorders, the interpretation of intermediate chromosomal values (e.g., mosaicism) remains less standardized. For example, a recent retrospective study showed that, in highly selected egg donation cohorts, the technology, protocols, and reporting strategy employed by the genetic service provider can significantly affect the euploidy, mosaicism, and, ultimately, live birth rates [47], highlighting the impact that the validation processes can have on the chance of successful outcomes following both IVF/ICSI and oocyte donation treatments.

This variability introduces inconsistencies in how results are interpreted, reported, and communicated to both clinicians and patients. Moreover, differences in threshold definitions and reporting practices can significantly influence clinical decision-making, leading to divergent approaches in embryo selection and patient counseling, and eventually affecting clinical outcomes in IVF cycles. Harmonization of these interpretive frameworks is, therefore, essential to improve consistency, transparency, and clinical utility across providers.

Regardless of the testing methodology used, it is critical that both analytical and clinical validity of the test are rigorously established prior to its implementation in a clinical setting. These validation steps must be undertaken for every newly developed PGT platform. Importantly, it should be noted that the optimal approach for clinical validation is a non-selection study design, in which the findings under investigation are only unblinded after embryo transfer. This helps minimize potential confounding factors, such as population or embryo selection biases, and ensures the reliability of the results.

The reliability of PGT technologies depends heavily on strict quality control procedures, proper equipment calibration, and the thorough validation of testing protocols, all of which must be demonstrated by the individual testing providers. To maintain high standards in test performance and result interpretation, genetic testing laboratories must operate under appropriate licensing and accreditation. These laboratories should comply with national regulations and align with internationally recognized quality systems, such as ISO 15189 [48], CLIA (Clinical Laboratory Improvement Amendments), and CAP (College of American Pathologists). Adherence to these standards ensures both technical competence and the accuracy, reliability, and consistency of results provided to clinicians and patients.

There is a critical need for prospective blinded clinical studies to establish the validity of emerging genomic findings; such studies are essential to objectively assess clinical utility, minimize bias, and ensure that new technologies meet rigorous standards before being applied in patient care. These validation studies should be conducted by qualified genetic professionals to demonstrate both the diagnostic accuracy and clinical utility of the tests used. Continuous monitoring and periodic revalidation are essential to ensure that diagnostic performance remains consistent over time, safeguarding the quality and reliability of genetic reports. In the absence of definitive evidence regarding the genetic or chromosomal status of the embryo, it is both prudent and ethically responsible to adopt a measured and proportionate approach. Reporting and clinical decision-making should be guided by scientific rigor, transparency, and a commitment to minimizing potential harm, particularly when the available data do not allow for unequivocal conclusions.

However, incorporating complementary analytical methods, such as qualitative NGS-based techniques (i.e., SNP analysis) alongside standard quantitative approaches, can significantly enhance diagnostic reliability, supporting aneuploidy calling. These additional validatory steps help clarify whether an intermediate copy number variation arises from a meiotic error (producing a below-threshold signal), which would typically indicate a uniform, non-viable aneuploid embryo, or from a mitotic event or technical artifact. This distinction is critical to avoid misclassification and to support more informed clinical decision-making.

## 10. Conclusions

While the performance of PGT for detecting monogenic conditions and uniform whole-chromosome aneuploidies is well established, the interpretation of more findings, such as mosaicism and segmental aneuploidy, continues to pose challenges and diverts from the main goal of PGT-A, which is deselecting embryos with uniform aneuploidies and no reproductive potential. These secondary findings from the PGT analysis would have required primary evidence of clinical validity and utility before being implemented in clinical practice. Looking ahead, both technical refinements and policy developments should prioritize the integration of validatory tools, such as SNP-based genotyping and determination of the biological or parental origin of abnormalities. These enhancements would improve the interpretation of genetic data, strengthen the accuracy of findings, and provide clearer insight into their potential clinical impact.

As PGT expands to include a broader range of genomic resolution, it is imperative that ethical considerations remain central to its application. Scientific progress must be balanced with responsible reproductive decision-making, ensuring that emerging technologies are applied in ways that are both clinically meaningful and socially responsible. Achieving this balance requires an ongoing, transparent, and collaborative dialog among reproductive medicine specialists, clinical geneticists, bioethicists, and regulatory authorities. Only through sustained interdisciplinary collaboration can the field of PGT progress toward ethically responsible, evidence-based, and patient-centered clinical application.
